# LncRNA BDNF‐AS inhibits proliferation, migration, invasion and EMT in oesophageal cancer cells by targeting miR‐214

**DOI:** 10.1111/jcmm.13558

**Published:** 2018-06-12

**Authors:** Huaying Zhao, Changying Diao, Xiaohui Wang, Yilin Xie, Yaqing Liu, Xianzheng Gao, Jing Han, Shenglei Li

**Affiliations:** ^1^ Department of Pathology The First Affiliated Hospital of Zhengzhou University Zhengzhou China

**Keywords:** BDNF‐AS, EMT, miR‐214, oesophageal cancer

## Abstract

This study was aimed at exploring the effect of lncRNA BDNF‐AS on cell proliferation, migration, invasion and epithelial‐to‐mesenchymal transition (EMT) of oesophageal cancer (EC) cells. The expression of BDNF‐AS and miR‐214 in tissue samples and cells was measured by qRT‐PCR. The targeted relationship between BDNF‐AS and miR‐214 was analysed by dual‐luciferase reporter assay. After cell transfection, the cell proliferation activity was assessed by MTS method, while the migrating and invading abilities were evaluated by transwell assay. LncRNA BDNF‐AS was remarkably down‐regulated, while miR‐214 was up‐regulated in EC tissues and cells in comparison with normal tissues and cells. Overexpression of BDNF‐AS significantly inhibited the abilities of cell proliferation, migration and invasion as well as the EMT processes of EC cells. The bioinformatics analysis and luciferase assay indicated that BDNF‐AS could be directly bound by miR‐214. Furthermore, overexpression of miR‐214 and BDNF‐AS exerted suppressive influence on EC cell multiplication, migration, invasion and EMT processes. LncRNA BDNF‐AS restrained cell proliferation, migration, invasion and EMT processes in EC cells by targeting miR‐214.

## 1 INTRODUCTION

Oesophageal cancer (EC), includes oesophageal adenocarcinoma (EAC) and oesophageal squamous cell carcinoma (ESCC), is the most common cancer in sixth place with a high incidence rate and one of the most fatal types of digestive tract malignant tumour as well.^1^ Although EAC often occurs in western countries almost particularly in histopathology, ESCC is one of the most mortal malignancies in Asian countries, such as China and Japan.^2^ Advances in chemoradiotherapy and surgery could prolong the survival time of patients with EC, but the majority of patients failed to undergo surgical resection because of the advanced stage. Moreover, the overall 5‐year survival rate of cases remains extremely low for lack of effective therapies.^3^ Over the past 20 years, studies have identified numerous carcinogenic and oncogenic proteins that are associated with the induction and treatment of EC. Nevertheless, it has not been recognized that molecular indicators of cellular deregulation were important in the EC. Improvements in EC disease diagnosis and therapy need a better understanding of the molecular pathways involved in EC development. Hence, it is essential for the development of biomarkers and therapeutic targets to identify the molecular mechanisms of EC.

Long non‐coding RNAs (lncRNAs) are novel ncRNAs incorporating over 200 nucleotides, which have been identified as crucial tumour modulators or potential cancer biomarkers.^4^ Recently, more and more studies have demonstrated that a large number of lncRNAs were either up‐regulated or down‐regulated in certain diseases and can epigenetically regulate coding DNAs to exert specific biological functions in human diseases.^5^ The epithelial mesenchymal transition (EMT) is a crucial biological process for the migration and invasion of tumour cells derived from epithelial cells. At present, the study on the mechanisms related to the EMT in the EC has become a major focus of research considering that most patients have local infiltration and distant metastasis at diagnosis.^6^ LncRNAs have been involved in multiple processes related to the EMT.^7^ For instance, Li et al found that lncRNA ATB, which was induced by TGF‐β, stimulated EMT through silencing miR‐200s and facilitated multiplication by balancing IL‐11 mRNA in hepatocellular carcinoma, thus promoting cancer metastasis.^8^ Moreover, lncRNA H19 promotes cell invasion and migration in pancreatic ductal adenocarcinoma by increasing HMGA2‐mediated EMT through antagonizing let‐7.^9^ LncRNA of brain‐derived neurotrophic factor antisense (BDNF‐AS) is a natural non‐coding antisense of neuronal transcriptional factor BDNF.^10^ BDNF‐AS was discovered to act as an adverse regulator of BDNF and has profound impacts in neuronal system.^11^ Although an increasing number of studies have revealed that BDNF could act as a cardinal oncogenic factor in human cancers, whether BDNF‐AS could also act as a functional regulator or biomarker in human cancer remains little known. Thus, in our study, we investigated the expression, prognostic potential and functional mechanisms of BDNF‐AS in EC.

MicroRNAs (miRNAs) are short non‐coding single‐stranded RNA molecules, which could regulate gene expression through base pairing with the 3′‐untranslated region (3′‐UTR) of target mRNAs, resulting in post‐transcriptional suppression or mRNA degradation.^12^ Growing findings have proved that microRNAs may play a critical role in carcinogenesis.^13^ Accumulating studies have certified that both up‐ and down‐regulation expression of miR‐214 can be causative for the progress of various malignant tumours, including pancreatic, hepatoblastoma, hepatocellular, lung, breast and cervical.^14^, ^15^ However, the role of miR‐214 in oesophageal cancer progression and the molecular mechanisms, in particular, EMT and subsequently invasion and metastasis, remains to be further investigated.

In this study, we present that BDNF‐AS inhibits the capacity of cell proliferation, invading and migrating in EC by regulating the EMT. We examined BDNF‐AS and miR‐214 mRNA levels in both EC biopsy specimens and in vitro EC cell lines and statistically analysed their expression in EC tissues and cells. Overexpression of BDNF‐AS was found to suppress cell proliferation, invasion and metastasis in vitro and suppress the EMT in EC cells. Further analysis demonstrated BDNF‐AS functioned as a miR‐214 sponge to positively regulate cell proliferation, metastasis and the EMT. These findings would provide novel insights into molecular mechanisms of EC development together with the new therapeutic approach in the future.

## 2 MATERIALS AND METHODS

### 2.1 Tissue samples and cell lines

Fifty‐four pairs of surgical primary EC tissues, corresponding adjacent normal tissues and non‐tumour samples, were obtained directly after surgical resection between the years of 2014 and 2016 at the First Affiliated Hospital of Zhengzhou University, China (Table S1). All biopsy specimens were directly frozen at −196°C (liquid nitrogen) and stored at −70°C before further treating. Ethical approval was ratified by the local Ethics Committee, and all patients provided written informed consent for the utilization of tissue samples in research.

Normal oesophageal epithelial cell line SHEE and eight EC cell lines (OE19, KYSE‐70, KYSE‐170, KYSE‐180, OE33, Eca‐109, TE‐1 and TE‐13) were furnished by the Bena Culture Collection (Beijing, China). Whole sequences of BDNF‐AS gene were magnified from a human cDNA library and inserted between BamHI and XhoI restriction sites of a pCDNA3/+ vector (Takara, China), resulting in a BDNF‐AS overexpression plasmid. Has‐miR‐214 mimic was purchased from the GenePharma (Shanghai, China). Primers were supplied by Applied Biosystems (CA, USA).

### 2.2 Cell culture and cell transfection

Cells were cultured in RPMI‐1640 medium (Thermo Fisher Scientific, USA) supplemented with 100 μg/mL streptomycin, 100 U/mL penicillin together with 10% foetal bovine serum (FBS, Thermo Fisher Scientific, USA), in a culture chamber with 5% CO_2_ at 37°C. Cells in the logarithmic growth phase were taken and cultured by paving them to six‐well plates. The constructs or oligos were transfected into OE19 and OE33 cell lines using Lipofectamine 2000 (Invitrogen, USA) on the manufacturer's instructions when they were 70%‐80% per cent fusion. The experiment was divided into five groups: untransfected group (control), negative control group (NC), BDNF‐AS transfection group (BDNF‐AS), miR‐214 mimic transfection group (miR‐214) and BDNF‐AS and miR‐214 mimic cotransfection group (BDNF‐AS + miR‐214).

### 2.3 Quantitative real‐time PCR (qRT‐PCR)

Total RNA was extracted from biopsy specimens or in vitro EC cell lines with the TRIzol reagent (Invitrogen, USA) following the manufacturer's protocol. RNA quantification was carried out using a NanoDrop™ 3000 Spectrophotometer (Thermo Fisher Scientific, USA), and complementary DNA (cDNA) was generated using the First Strand cDNA Synthesis kit (Thermo, USA). QRT‐PCR was conducted using a Platinum SYBR Super Mix (Stratagene, USA) according to the manufacturer's protocol. The brief reaction was as follows: 95°C for 5 minutes, then 40 cycles of 95°C for 15 seconds followed by 60°C for 3 seconds and finally 72°C for 30 seconds. The change in transcript abundance was calculated using the mastercycler sample analysis software. The endogenous control for miR‐214 was U6 RNA. Others tested in this study were standardized to GAPDH. Primers are reported in Table 1.

**Table 1:** Primer sequences used in qRT‐PCR

Gene	Primer sequence
miR‐214	F: 5′‐AGCATAATACAGCAGGCACAGAC‐3′
	R: 5′‐AAAGGTTGTTCTCCACTCTCTCAC‐3′
U6	F: 5′‐CTGGCTTCGGCAGCACA‐3′
	R: 5′‐AACGCTTCACGAATTTGCGT‐3′
E‐cadherin	F: 5′‐TGGACAGGGAGGATTTTGAG‐3′
	R: 5′‐ACCTGAGGCTTTGGATTCCT‐3′
N‐cadherin	F: 5′‐CCACAGCTCCACCATATGACT‐3′
	R: 5′‐CCCCAGTCGTTCAGGTAATC‐3′
Vimentin	F: 5′‐AGTGCCTGGAACGTCAGATG‐3′
	R: 5′‐CAGCAGCTTCCTGTAGGTGG‐3′
GAPDH	F:5′‐CGGATTTGGTCGTATTGGG‐3′
	R:5′‐TGCTGGAAGATGGTGATGGGATT‐3′

F: forward, R: reverse.

### 2.4 MTS assay

Logarithmic growth phase cells were suspended and inoculated in 96‐well plate with the density of 3000 cells/well in 100 μL medium. 0 hour, 24 hours, 48 hours, 72 hours and 96 hours after adherence, additional 20 μL MTS reagent (500 μg/mL) was put into the wells of 96‐well plate and cells incubated of 2.5 hours at 37°C for a minimum. Absorbance values were determined at 492 nm using a microplate reader (ELx 800; BioTek Instruments Inc., Winooski, VT, USA). Each experiment was conducted in triplicate.

### 2.5 Transwell migration and invasion assay

Cell invasion assay was carried out using 24‐well Matrigel chambers containing 12 μL Matrigel‐coated membranes with even covered with small room floor. Each cell suspension in serum‐free medium RPMI1640 was counted by microscope. A medium with 10% FBS (600 μL) was added to the lower wells. Each group cells (1 × 10^4^ cells in 200 μL of medium without serum cell suspension) were added into the upper transwell chambers (8‐mm pore size, BD, USA). Matrigel chambers were incubated at 37°C, the volume fraction of 5% CO_2_ incubator for 24 hours. Migration assays were performed in a similar mode using chambers without the Matrigel coating. After incubation, the cells on the upper membrane surface were wiped away using a cotton swab and the cells from the bottom membrane surface were fixed with methanol, stained with 0.1% crystal violet. Then, five areas were randomly chosen in each chamber.

### 2.6 Western blot

OE19 and OE33 cells were collected and lysed with SDS lysis buffer (Promega, USA). After determining the concentration of protein with the BCA assay, the equal amount of protein samples was separated by 10% SDS‐PAGE gel (BD, USA) and then transferred to PVDF membranes. After being disposed with 5% skimmed milk, these membranes were then blocked at room temperature for 1 hour followed by being washed 10 minutes for three times. For primary antibody incubation, PVDF membranes were treated with a rabbit antibody against N‐cadherin, vimentin, E‐cadherin and GADPH (1:200, Proteintech, USA) for all night at 4°C. After primary antibody treatment, membranes were washed 10 minutes for three times. Afterwards, membranes were incubated with the secondary horseradish peroxidase‐conjugated antibody (ROCKLAND, USA) for 1 hour at room temperature avoiding light. Membranes were washed once more 10 minutes for three times. The blots were assessed by an enhanced chemiluminescence (Amersham Biosciences, USA) according to the manufacturer's protocol.

### 2.7 Luciferase reporter assay

OE19 cells and OE33 cells were cotransfected by firefly luciferase reporter plasmid (BDNF‐AS‐WT, BDNF‐AS‐MUT,) and a renilla luciferase vector (pRL‐SV40, Promega) plus negative control and small RNAs (NC, miR‐214) using Lipofectamine 2000 (Invitrogen, USA). Experiments were carried out at least 3 times. The luciferase activities from pGL3‐control‐derived plasmids were standardized to renilla luciferase activity from pRL‐SV40 by the luciferase assay system (Promega, USA). After post‐transfection for 48 hours, luciferase activity was evaluated in the harvested cells using the Multimode Detector reporter assay system (Beckman Coulter, WI, USA).

### 2.8 Statistical analysis

Data were expressed as the mean ± SD (standard deviation). All data were calculated using SPSS 21.0 (SPSS, USA). The comparison between two independent groups was tested by adopting the Student's *t* test. The comparison of mean standard between multiple groups was carried out using one‐way ANOVA method. The SNK‐q test was used for intragroup comparison. Chi‐square test was used to estimate the clinicopathological features of EC. *P*‐values <.05 were deemed statistically significant.

## 3 RESULTS

### 3.1 Expression levels of BDNF‐AS and miR‐214 in EC tissue samples and cell lines

**Figure 1:**
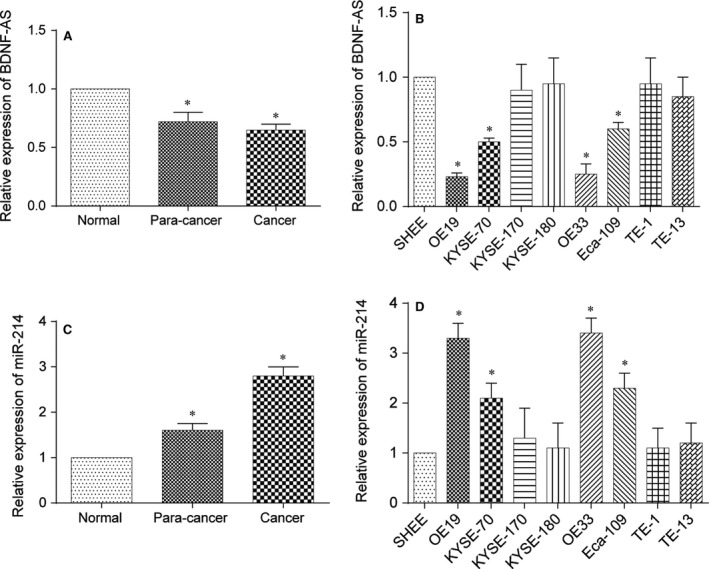
Differential expression of BDNF‐AS and miR‐214 in tissues and cell lines was surveyed by qRT‐PCR. (A) Expression levels of BDNF‐AS in patients’ tissue samples. *P < .05 compared to the non‐tumour tissues group. (B) Expression levels of BDNF‐AS in the normal cell line and EC cell lines. *P < .05 compared to the SHEE group. (C) Expression levels of miR‐214 in patients’ tissue samples. *P < .05 compared to the non‐tumour tissues group. (D) Expression levels of miR‐214 in the normal cell line and EC cell lines. *P < .05 compared to the SHEE group

QRT‐PCR was used to measure BDNF‐AS and miR‐214 expression levels in clinical samples and cell lines, which were normalized to U6. Lower expression of BDNF‐AS was observed in the EC tissues and the corresponding adjacent normal tissues than the non‐tumour tissues (*P *< .05, Figure 1A). In addition, qRT‐PCR was utilized to gauge expression levels of BDNF‐AS in EC cell lines (including OE19, KYSE‐70, KYSE‐170, KYSE‐180, OE33, Eca‐109, TE‐1 and TE‐13) and normal oesophageal epithelial cells (SHEE). Notably, BDNF‐AS expression was significantly down‐regulated in cell lines including OE19, KYSE‐70, OE33 and Eca‐109 compared with SHEE (*P *< .05, Figure 1B). Inversely, the miR‐214 expression in EC tissues and the corresponding adjacent normal tissues was significantly aggrandized compared with non‐tumour tissues (*P *< .05, Figure 1C). In addition, the expression levels of miR‐214 in OE19, KYSE‐70, OE33 and Eca‐109 cell lines were remarkably exceeded those in SHEE (*P* < .05,Figure 1D). The OE19 and OE33 cell lines were used for subsequent experiments for their most significant differences.

### 3.2 Overexpression of BDNF‐AS restrained cell growth, migration and invasion of EC cells

**Figure 2:**
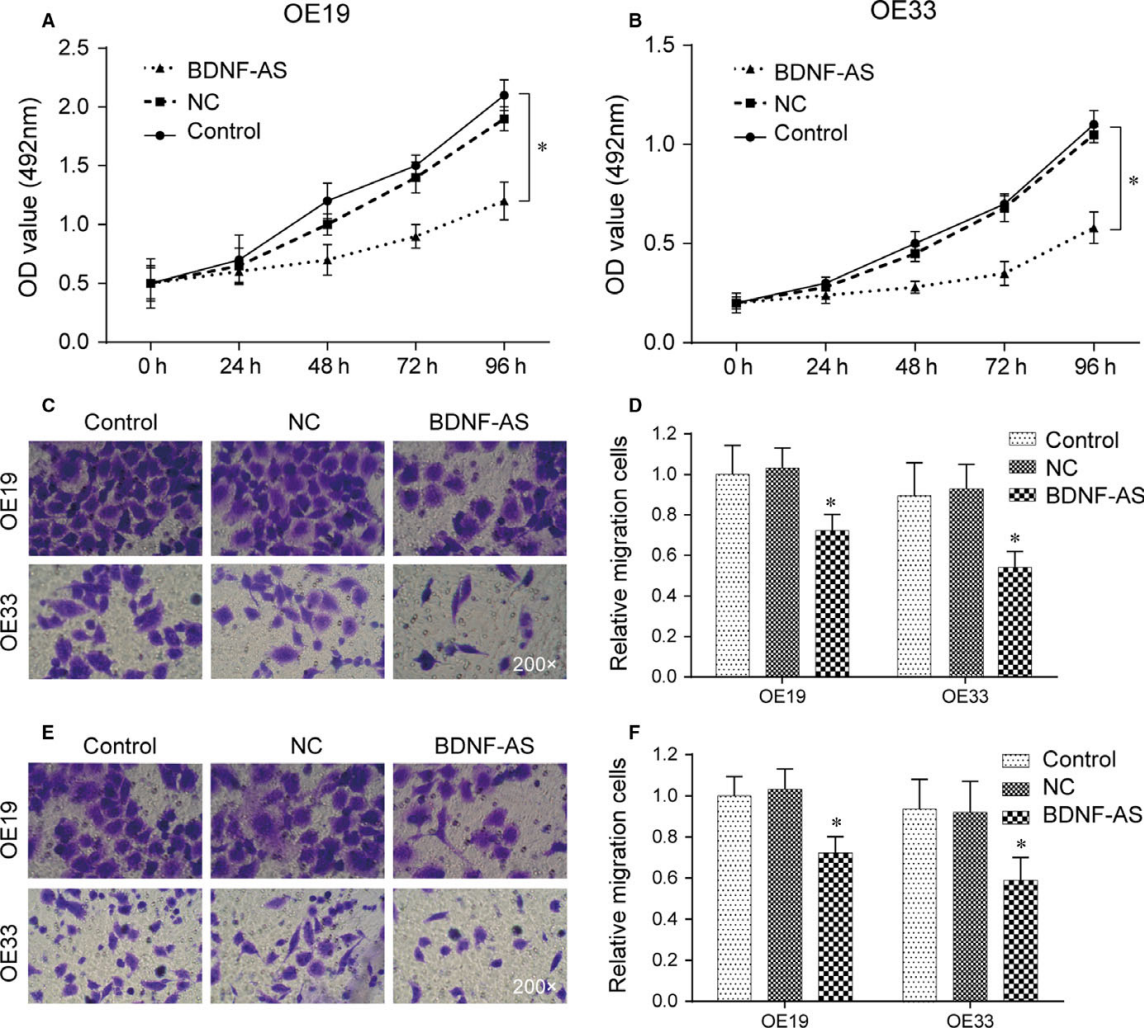
Effects of BDNF‐AS on cell proliferation, migration and invasion ability. (A‐B) Cell proliferation ability of OE19/OE33 in control group, NC group and BDNF‐AS transfection group was detected by MTS assay. *P < .05 compared to the control group. (C‐F) After 48 h culture in transwell chamber, the number of migration and invasion cells was calculated under high‐power microscope. *P < .05 compared to the control group

The growth curves of EC cells in untransfected group (Control), negative control group (NC) and BDNF‐AS transfection group (BDNF‐AS) were drawn at absorbance of 492 nm measured by ELISA (Figure 2A,B). The MTS results displayed that the transfection of BDNF‐AS inhibited cell proliferation ability of OE19 and OE33 in vitro (*P* < .05).

After 48 hours culture in transwell chambers, the number of EC cells traversing the basement membrane was calculated under an inverted phase microscope. Compared with control groups, overexpression of BDNF‐AS displayed impeded migration in both OE19 and OE33 cells (*P* < .05, Figure 2C,D). Similarly, invasion of OE19 and OE33 cells was reduced following overexpression of BDNF‐AS (*P* < .05, Figure 2E,F). Furthermore, no significant change was found out between untransfected control group and NC group (*P* > .05). Overall, these results demonstrate that overexpression of BDNF‐AS could suppress the migration and invasive ability of EC cells in vitro.

### 3.3 Overexpression of BDNF‐AS suppressed the EMT in EC cells

**Figure 3:**
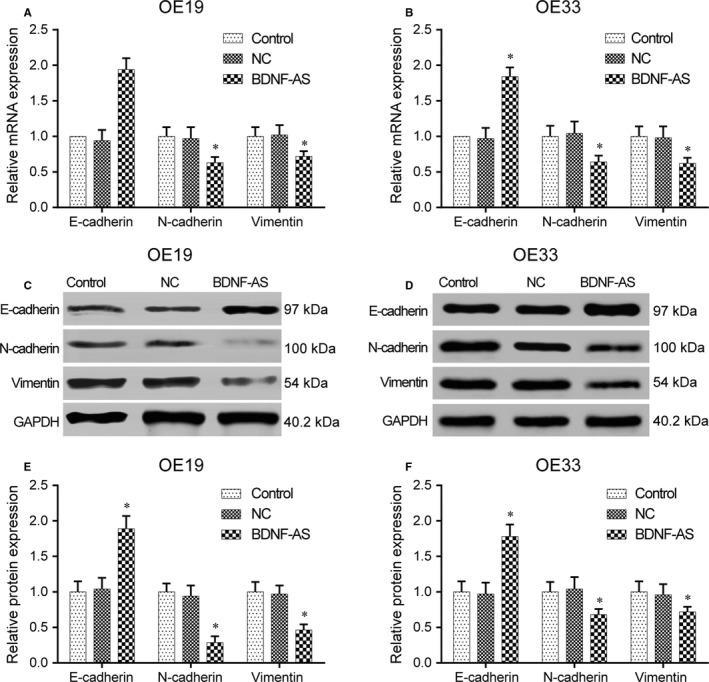
Impacts of BDNF‐AS on expression of EMT‐related genes (A‐B) BDNF‐AS plasmids and negative control vectors were transfected into the primary EC cells, respectively. After incubation for 48 h, EMT‐related mRNA expression levels were measured by real‐time qPCR. *P < .05 compared to the control group. (C‐F) After transfection for 48 h, expression levels of EMT‐related proteins were detected by Western blot. *P < .05 compared to the control group

Next, we examined the quantity of the expression of EMT markers to explore the effect of lncRNA BDNF‐AS on the EMT in EC. Results of qRT‐PCR revealed that overexpression of BDNF‐AS in OE19 and OE33 cells up‐regulated E‐cadherin mRNA and down‐regulated N‐cadherin and vimentin mRNA compared to the untransfected control group and negative control transfection group (*P* < .05, Figure 3A,B). Similarly, results of Western blot indicated that the E‐cadherin protein expression level was up‐regulated, while the N‐cadherin and vimentin protein expression levels were significantly down‐regulated in the transfection group (*P* < .05, Figure 3C‐F), indicating BDNF‐AS inhibited the EMT in EC.

### 3.4 LncRNA BDNF‐AS directly bound to miR‐214

**Figure 4:**
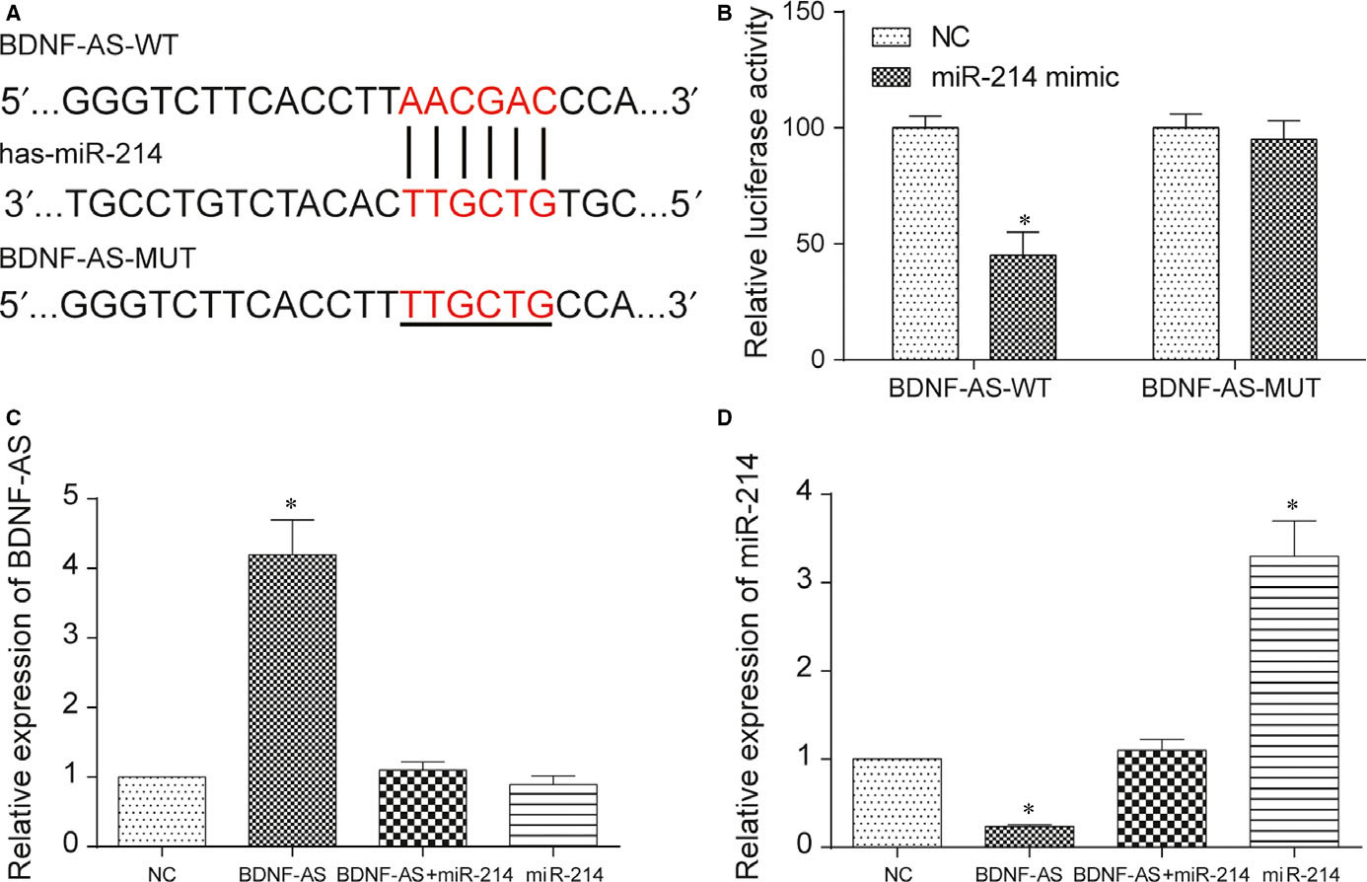
LncRNA BDNF‐AS directly bound with miR‐214. (A) Bioinformatics analysis revealed BDNF‐AS contains binding sequences complementary to the binding sites of miR‐214, and the binding site mutation is underlined. (B) Dual‐luciferase reporter assay verified the targeting relationship between miR‐214 and BDNF‐AS. (C) The expression of BDNF‐AS was evaluated by qRT‐PCR after cells transfected with BDNF‐AS, miR‐214 or BDNF‐AS+miR‐214. (D) The expression of miR‐214 was measured by qRT‐PCR after cells treated with BDNF‐AS, miR‐214 or BDNF‐AS+miR‐214. *P < .05 compared to the NC group

To explore interaction between lncRNA BDNF‐AS and miR‐214, we compared the sequence of BDNF‐AS with that of miR‐214 by referring the miRcode database (http://www.mircode.org/). We noticed that there was an expected binding site of miR‐214 in BDNF‐AS (Figure 4A). A dual‐luciferase reporter assay was used to corroborate the function of these binding sites. The wild‐type BDNF‐AS sequence (BDNF‐AS‐WT) or mutant sequence (BDNF‐AS‐MUT) was cotransfected with miR‐214 mimics or miR‐NC into cells. The luciferase activity of the luciferase reporter containing BDNF‐AS‐WT was significantly lower in the overexpression of miR‐214 group (*P* < .05), but indistinguishable in the reporter containing BDNF‐AS‐MUT (*P* > .05, Figure 4B). These data indicated that lncRNA BDNF‐AS could directly sponge miR‐214. As shown in Figure 4C, the expression of BDNF‐AS increased in the BDNF‐AS group, while miR‐214 had no obvious effect on the expression of BDNF‐AS (*P* < .05). According to the expression of miR‐214, BDNF‐AS could suppress miR‐214 expression and the expression of miR‐214 enlarged after cells were overexpressed miR‐214 (Figure 4D, *P* < .05).

### 3.5 Effects of cotransfection of BDNF‐AS and miR‐214 on proliferation, migration and invasion of OE19 and OE33 cells

**Figure 5:**
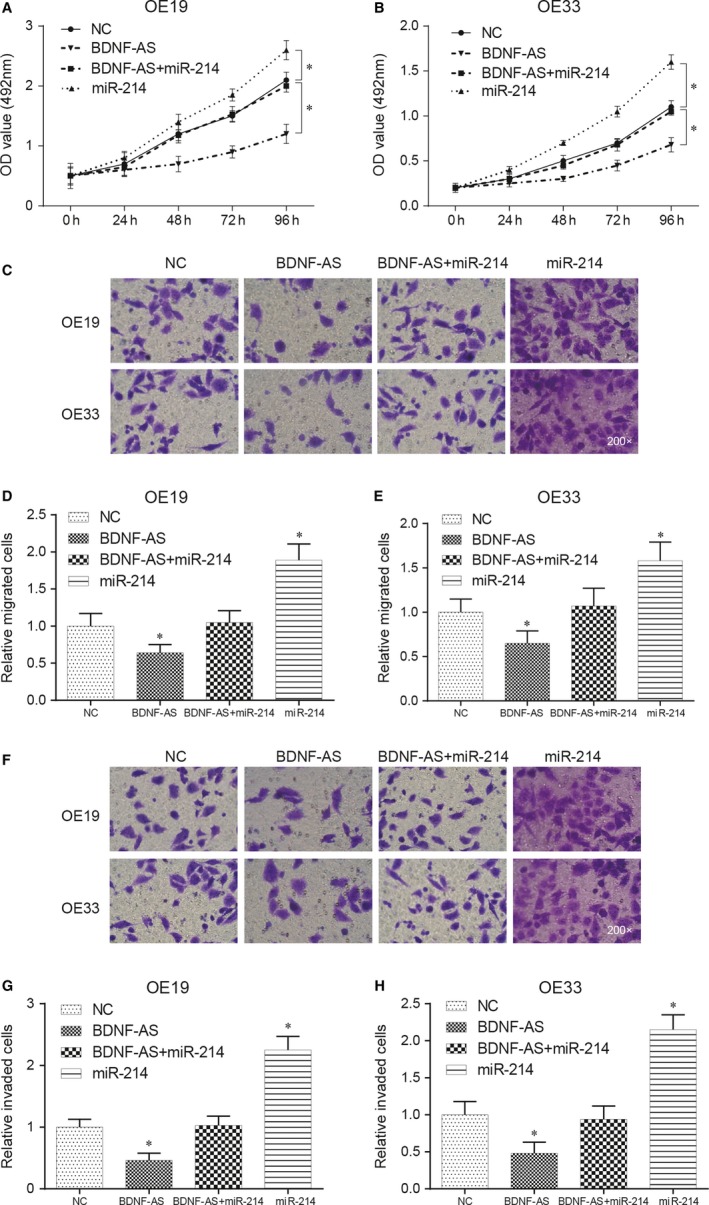
Effects of miR‐214 on proliferation, migration and invasion of OE19 and OE33 cells (A‐B) After transfection, effects of cotransfection of BDNF‐AS and miR‐214 on cell proliferation were analysed by MTS cell growth curves. *P < .05 compared to the NC group. (C‐H) The number of migration/invasion cells was detected by transwell migration/invasion assay. Effects of cotransfection of BDNF‐AS and miR‐214 on cell migration/invasion ability were analysed. *P < .05 compared to the NC group

Results of MTS assay showed that transfection of BDNF‐AS could obstruct the cell growth of EC cells, and the transfection of miR‐214 mimic promoted the proliferation activity of EC cells in vitro (*P* < .05). In addition, no significant change is found in proliferation activity after cotransfection of BDNF‐AS and miR‐214 mimic (*P* > .05, Figure 5A,B).

Upon the transwell migration assay result, the number of migration cells in BDNF‐AS transfection group was much less than that in the NC group (*P* < .05), and migration cells in miR‐214 mimic transfection group significantly overtopped the NC group (*P* < .05, Figure 5C‐E). Analogously, transwell invasion assay shared similar results, overexpression of BDNF‐AS inhibited cell invasion significantly, while overexpression of miR‐214 promoted cell invasion observably (*P* < .05, Figure 5F‐H). There was no significant difference in both migration and invasion numbers between BDNF‐AS + miR‐214 mimic cotransfection group and the NC group (*P* > .05).

### 3.6 Effects of cotransfection of BDNF‐AS and miR‐214 on expression of EMT‐related factors in EC cells

**Figure 6:**
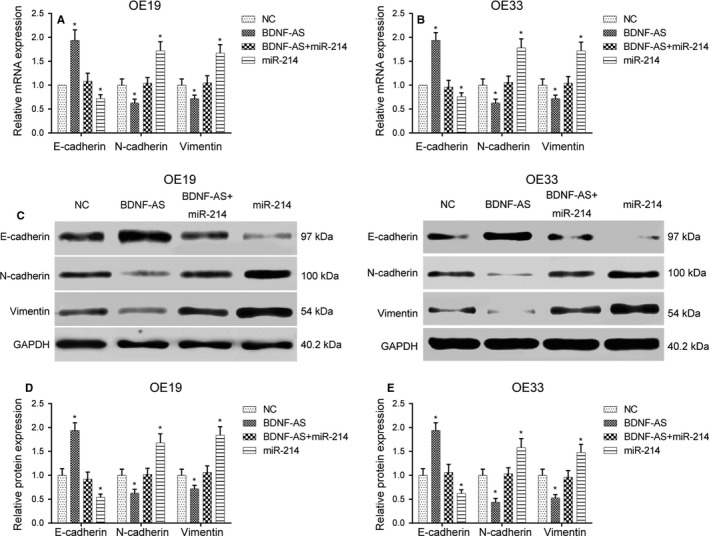
Effects of miR‐214 on expression of EMT‐related factors in EC cells (A‐B) mRNA expression levels of EMT‐related genes were detected by qRT‐PCR after transfection. *P < .05 compared to the NC group. (C‐E) The levels of protein expression of EMT‐related genes were detected by Western blot. *P < .05 compared to the NC group

To explore how lncRNA BDNF‐AS and miR‐214 regulated the EMT in EC, the expression levels of EMT‐related mRNA and protein were, respectively, measured by qRT‐PCR and Western blot assays. Overexpression of miR‐214 EC cells down‐regulated E‐cadherin mRNA and protein expression and up‐regulated N‐cadherin and vimentin expression in contrast to the NC group (*P* < .05). Nevertheless, there were no noteworthy differences between BDNF‐AS + miR‐214 mimic cotransfection group and the NC group (*P* > .05, Figure 6).

## 4 DISCUSSION

Human oesophageal cancer (EC) ranks ninth of occurrence and sixth as the leading cause of cancer mortality, which occurs around the world in a varying geographic distribution and influences male more than female.^1^ In spite of the development of miscellaneous therapies, the prognosis of the patient with EC remains poor, indicating the limitations of conventional treatment, which motivates us to investigate an innovative treatment for ECs.^16^ Current risky therapeutic strategies may be improved due to the recognition of new prognostic biological markers for EC, which helps ameliorate unfavourable clinicopathological characteristics and shorter survival for patients.^3^ In this study, we first described that lncRNA BDNF‐AS expression in EC positively correlated with overall survival of patients. The results of our study demonstrated that BDNF‐AS, a miRNA‐214 sponge, was significantly down‐regulated in EC tissues and cell lines compared with normal specimens, and overexpression of BDNF‐AS could inhibit the ability of cell growth, migration, invasion and the EMT process in EC cells. The discovery of the tumour suppressive role of lncRNA BDNF‐AS is not only of great significance in mechanism and function of the study on lncRNA function in human cancers, but may also accelerate the programs of new treatment.

Increasingly, lncRNA elements co‐ordinate the molecular integration of the information flow by sending the correct transcripts at the right time to the correct location.^17^ Over the past few years, some studies have revealed that 18% of the protein‐coding genes that produce lncRNAs are associated with cancers, whereas for all human protein‐coding genes, the ratio is only 9%.^18^ Because of their great significance in the gene expression regulation, lncRNAs are confirmed to be associated with diversified cellular functions including cell proliferation, apoptosis, migration, invasion and differentiation and existed in various physiological and pathological processes.^19^ Thus, it is of paramount importance to identify and investigate the cancer‐associated lncRNAs, which helps better understand the roles of lncRNAs in cancer progression and develop novel therapeutic targets. In this study, several key observations we made were related with lncRNAs in EC. Firstly, we focused on, a widely expressed but previously unstudied in EC lncRNA, BDNF‐AS, as being down‐regulated in EC tissues and cell lines. Then, we discovered that overexpression of BDNF‐AS exerted inhibitory influence on EC cell proliferation, migration and invasion in vitro. In addition, we also paid our attention to the EMT process, which was closely associated with tumour metastasis.

EMT is the process which usually occurs in the period of the embryonic development and organogenesis.^20^ However, abnormal activation of the EMT facilitates the pathogenesis of tumour, thus inducing metastasis.^21^ LncRNAs play an important role in inducing the EMT in various types of tumour.^22^ Our study indicated that lncRNA BDNF‐AS inhibited the EMT in EC cells. The epithelial marker E‐cadherin, the mesenchymal marker N‐cadherin and vimentin are associated with the EMT.^23^, ^24^ In this assay, we explored the effects of overexpression of BDNF‐AS on the EMT process. According to the results of qRT‐PCR and Western blot, the expression quality of E‐cadherin was up‐regulated, while the expression of N‐cadherin and vimentin was down‐regulated with the treatment of BDNF‐AS overexpression, indicating the inhibition role of BDNF‐AS in the EMT process.

MicroRNAs (miRNAs) were discovered to be dysregulated in certain human cancers including EC.^25^ Growing evidence has confirmed that miRNAs can play a vital role in the regulation of development, differentiation, proliferation, metastasis and apoptosis.^26^, ^27^, ^28^ In our study, we examined the expression level of miR‐214 in EC tissues and cell lines and found the up‐regulation of miR‐214 in EC. Inspired by the preceding evidence and “competitive endogenous RNAs” regulatory network that lncRNA BDNF‐AS may be involved in this regulatory loop, we speculated that BDNF‐AS and miR‐214 may also act as ceRNAs in EC. Both bioinformatics analysis and luciferase assays were employed to affirm the direct binding sites of miR‐214 and BDNF‐AS. We discovered miR‐214 could form complementary base pairing with BDNF‐AS as expected and found miR‐214 and BDNF‐AS have the opposite functions. All in all, these results are in accordance with our hypothesis and manifest that BDNF‐AS may interact with miR‐214. In addition, cotransfection of BDNF‐AS and miR‐214 further confirmed the effects of their interaction on cell proliferation, migration and invasion.

These findings underpin the potential of systemic miR‐214 and BDNF‐AS therapy as a promising treatment for EC and in prevention of cancer metastasis. However, it remains largely unknown what are the downstream targets of miR‐214, and further studies are expected to explore the mechanism of miR‐214 and its downstream as well as involved signalling pathways, to expand the understanding of the target treatment of EC. In addition, in vivo experiments were taken into consideration in our further study.

## 5 CONCLUSION

In summary, our study focuses on the mechanism of EC progression, including cell proliferation, invasion and metastasis. Pivotal evidence observed in our study could support the hypothesis that the overexpression of the antioncogenic lncRNA BDNF‐AS is relevant to both clinic and function in the progression of EC, which inhibits cell proliferation, migration, invasion and the EMT process. In addition, the increased expression of miR‐214 has been associated with poor prognosis. Further analysis demonstrates BDNF‐AS functions as a miR‐214 sponge to facilitate cell proliferation, migration and invasion in the EC. Taken together, all these findings suggested that BDNF‐AS has essential effects on the pathogenesis of EC. Understanding the specific role of BDNF‐AS in the development and progression of the EC will provide a novel diagnostic marker and efficacious therapeutic strategies for EC treatment.

## CONFLICT OF INTEREST

The authors confirm that there is no conflict of interests.

## AUTHOR CONTRIBUTIONS

Huaying Zhao and Changying Diao conceived and designed the research. Xiaohui Wang, Yilin Xie, Xianzheng Gao and Shenglei Li analysed and interpreted the data. Changying Diao, Yaqing Liu, Xiaohui Wang and Jing Han performed statistical analysis. Huaying Zhao drafted the manuscript. All authors critically revised the manuscript and approved the final version of the manuscript.

## ETHICAL APPROVAL

This study was approved by the ethical committee of the First Affiliated Hospital of Zhengzhou University, and all participants signed the informed consent.

## INFORMED CONSENT

Informed consent has been obtained from each patient or subject after full explanation of the purpose and nature of all procedures used.

## References

[1] Torre LA , Bray F , Siegel RL , et al. Global cancer statistics, 2012. CA Cancer J Clin. 2015;65:87‐108. 25651787 10.3322/caac.21262

[2] Jemal A , Bray F , Center MM , et al. Global cancer statistics. CA Cancer J Clin. 2011;61:69‐90. 21296855 10.3322/caac.20107

[3] Li X , Wu Z , Mei Q , et al. Long non‐coding rna hotair, a driver of malignancy, predicts negative prognosis and exhibits oncogenic activity in oesophageal squamous cell carcinoma. Br J Cancer. 2013;109:2266‐2278. 24022190 10.1038/bjc.2013.548 PMC3798955

[4] Sugihara H , Ishimoto T , Miyake K , et al. Noncoding rna expression aberration is associated with cancer progression and is a potential biomarker in esophageal squamous cell carcinoma. Int J Mol Sci. 2015;16:27824‐27834. 26610479 10.3390/ijms161126060 PMC4661918

[5] Li X , Wu Z , Fu X , Han W . Long noncoding rnas: insights from biological features and functions to diseases. Med Res Rev. 2013;33:517‐553. 22318902 10.1002/med.21254

[6] Xu F , Zhang J . Long non‐coding rna hotair functions as mirna sponge to promote the epithelial to mesenchymal transition in esophageal cancer. Biomed Pharmacother. 2017;90:888‐896. 28441714 10.1016/j.biopha.2017.03.103

[7] Sarkar D , Leung EY , Baguley BC , Finlay GJ , Askarian‐Amiri ME . Epigenetic regulation in human melanoma: past and future. Epigenetics. 2015;10:103‐121. 25587943 10.1080/15592294.2014.1003746 PMC4622872

[8] Li W , Kang Y . A new lnc in metastasis: long noncoding rna mediates the prometastatic functions of tgf‐beta. Cancer Cell. 2014;25:557‐559. 24823634 10.1016/j.ccr.2014.04.014 PMC4091806

[9] Scherer S , Hammal Z , Yang Y , Morency LP , Cohn JF . Dyadic behavior analysis in depression severity assessment interviews. Proc ACM Int Conf Multimodal Interact. 2014;2014:112‐119. 28345076 10.1145/2663204.2663238 PMC5365085

[10] Modarresi F , Faghihi MA , Lopez‐Toledano MA , et al. Inhibition of natural antisense transcripts in vivo results in gene‐specific transcriptional upregulation. Nat Biotechnol. 2012;30:453‐459. 22446693 10.1038/nbt.2158 PMC4144683

[11] Zheng X , Lin C , Li Y , et al. Long noncoding rna bdnf‐as regulates ketamine‐induced neurotoxicity in neural stem cell derived neurons. Biomed Pharmacother. 2016;82:722‐728. 27470416 10.1016/j.biopha.2016.05.050

[12] He L , Hannon GJ . Micrornas: small rnas with a big role in gene regulation. Nat Rev Genet. 2004;5:522‐531. 15211354 10.1038/nrg1379

[13] Niemoeller OM , Niyazi M , Corradini S , et al. Microrna expression profiles in human cancer cells after ionizing radiation. Radiat Oncol. 2011;6:29. 21453501 10.1186/1748-717X-6-29 PMC3079656

[14] Wang F , Liu M , Li X , Tang H . Mir‐214 reduces cell survival and enhances cisplatin‐induced cytotoxicity via down‐regulation of bcl2 l2 in cervical cancer cells. FEBS Lett. 2013;587:488‐495. 23337879 10.1016/j.febslet.2013.01.016

[15] Peng RQ , Wan HY , Li HF , et al. Microrna‐214 suppresses growth and invasiveness of cervical cancer cells by targeting udp‐n‐acetyl‐alpha‐d‐galactosamine: polypeptide n‐acetylgalactosaminyltransferase 7. J Biol Chem. 2012;287:14301‐14309. 22399294 10.1074/jbc.M111.337642 PMC3340176

[16] Rizk NP , Ishwaran H , Rice TW , et al. Optimum lymphadenectomy for esophageal cancer. Ann Surg. 2010;251:46‐50. 20032718 10.1097/SLA.0b013e3181b2f6ee

[17] Mercer TR , Mattick JS . Structure and function of long noncoding rnas in epigenetic regulation. Nat Struct Mol Biol. 2013;20:300‐307. 23463315 10.1038/nsmb.2480

[18] Wapinski O , Chang HY . Long noncoding rnas and human disease. Trends Cell Biol. 2011;21:354‐361. 21550244 10.1016/j.tcb.2011.04.001

[19] Gibb EA , Vucic EA , Enfield KS , et al. Human cancer long non‐coding rna transcriptomes. PLoS ONE. 2011;6:e25915. 21991387 10.1371/journal.pone.0025915 PMC3185064

[20] Li B , Han Q , Zhu Y , et al. Down‐regulation of mir‐214 contributes to intrahepatic cholangiocarcinoma metastasis by targeting twist. FEBS J. 2012;279:2393‐2398. 22540680 10.1111/j.1742-4658.2012.08618.x

[21] Ansieau S , Courtois‐Cox S , Morel AP , Puisieux A . Failsafe program escape and emt: a deleterious partnership. Semin Cancer Biol. 2011;21:392‐396. 21986518 10.1016/j.semcancer.2011.09.014

[22] Jiang C , Li X , Zhao H , Liu H . Long non‐coding rnas: potential new biomarkers for predicting tumor invasion and metastasis. Mol Cancer. 2016;15:62. 27686732 10.1186/s12943-016-0545-z PMC5043609

[23] Rhim AD , Mirek ET , Aiello NM , et al. Emt and dissemination precede pancreatic tumor formation. Cell. 2012;148:349‐361. 22265420 10.1016/j.cell.2011.11.025 PMC3266542

[24] Satelli A , Li S . Vimentin in cancer and its potential as a molecular target for cancer therapy. Cell Mol Life Sci. 2011;68:3033‐3046. 21637948 10.1007/s00018-011-0735-1 PMC3162105

[25] Lin C , Liu A , Zhu J , et al. Mir‐508 sustains phosphoinositide signalling and promotes aggressive phenotype of oesophageal squamous cell carcinoma. Nat Commun. 2014;5:4620. 25099196 10.1038/ncomms5620

[26] Fan Z , Cui H , Xu X , et al. Mir‐125a suppresses tumor growth, invasion and metastasis in cervical cancer by targeting stat3. Oncotarget. 2015;6:25266‐25280. 26389681 10.18632/oncotarget.4457 PMC4694830

[27] Gong Y , He T , Yang L , et al. The role of mir‐100 in regulating apoptosis of breast cancer cells. Sci Rep. 2015;5:11650. 26130569 10.1038/srep11650 PMC4486956

[28] Ding Z , Jian S , Peng X , et al. Loss of mir‐664 expression enhances cutaneous malignant melanoma proliferation by upregulating plp2. Medicine (Baltimore). 2015;94:e1327. 26287415 10.1097/MD.0000000000001327 PMC4616445

