# Correction to “LncRNA BDNF‐AS inhibits proliferation, migration, invasion and EMT in oesophageal cancer cells by targeting miR‐214”

**DOI:** 10.1111/jcmm.18273

**Published:** 2024-05-22

Zhao H, Diao C, Wang X, et al. LncRNA BDNF‐AS inhibits proliferation, migration, invasion and EMT in oesophageal cancer cells by targeting miR‐214. *J Cell Mol Med*. 2018;22:3729‐3739.

In Zhao et al. (JCMM‐10‐2017‐078), the author uploaded the wrong version of Figure 5C–H due to technical error during image preparation. The whole correct figure is shown below. The authors confirm all results and conclusions of this article remain unchanged.

**FIGURE 5:**
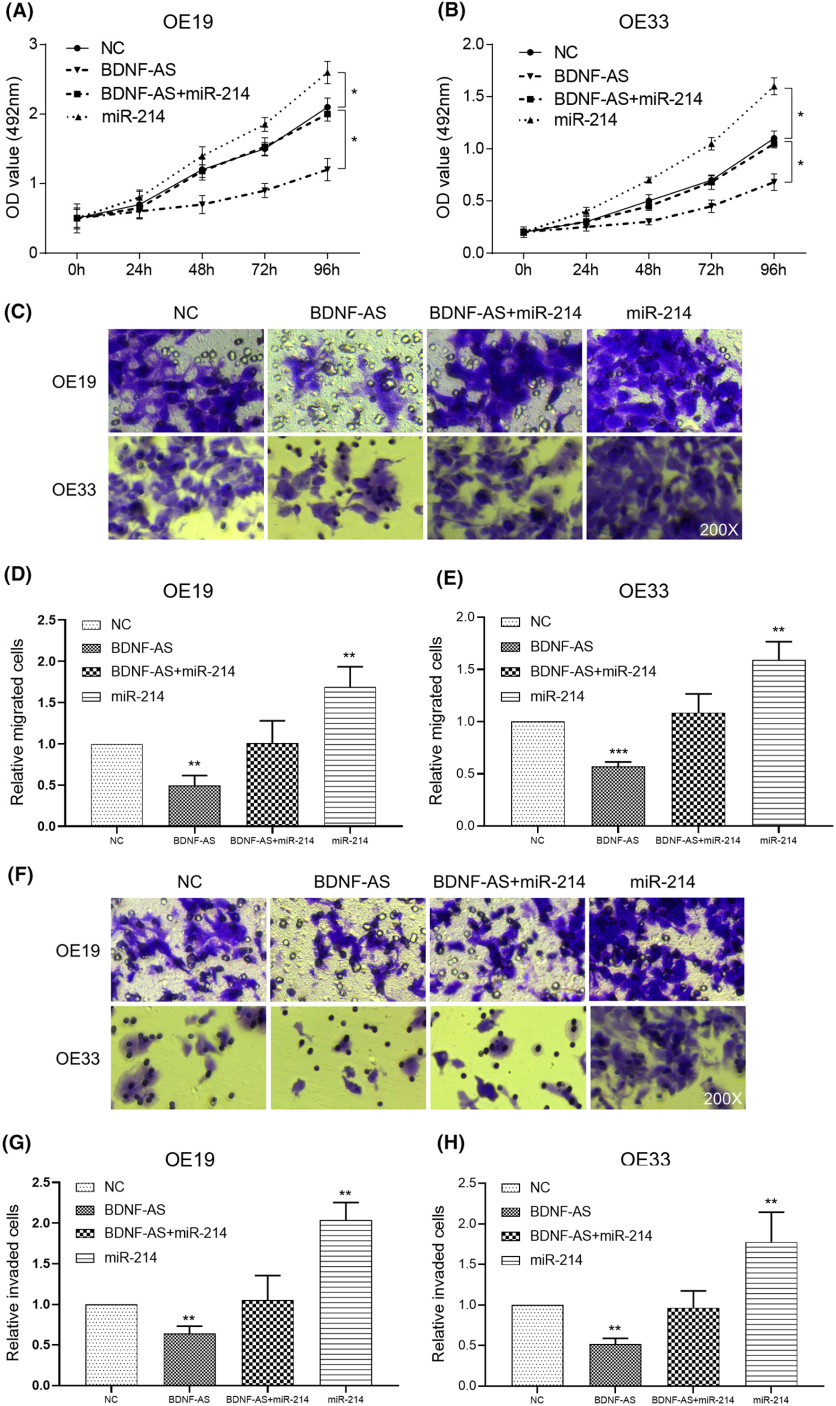
Effects of miR‐214 on proliferation, migration and invasion of OE19 and OE33 cells. (A, B) After transfection, effects of co‐transfection of BDNF‐AS and miR‐214 on cell proliferation were analysed by MTS cell growth curves. *p < 0.05 compared to the NC group. (C–H) The number of migration/invasion cells was detected by Transwell migration/invasion assay. Effects of co‐transfection of BDNF‐AS and miR‐214 on cell migration/invasion ability were analysed. **p < 0.01 and ***p < 0.001 compared to the NC group.

