# Supplementary material for: LncRNA BDNF‐AS inhibits proliferation, migration, invasion and EMT in oesophageal cancer cells by targeting miR‐214
Source: J Cell Mol Med. 2018 Jun 12;22(8):3729–39. doi: 10.1111/jcmm.13558 (PMC6050505; doi:10.1111/jcmm.13558)
Supplement: Supplementary file 1 [file JCMM-22-3729-s001.docx]

**Supplementary Table S1.** Relationship between BDNF-AS expression and clinicopathological features

| Clinicopathological feature | N | BDNF-AS expression | | χ^2^ | *P* value |
| --- | --- | --- | --- | --- | --- |
|  |  | low | high |  |  |
| Age/year |  |  |  | 0.307 | 0.580 |
| ＜60 | 22 | 10 | 12 |  |  |
| ≥60 | 32 | 17 | 15 |  |  |
| Histological grade |  |  |  | 0.384 | 0.825 |
| Ⅰ | 5 | 3 | 2 |  |  |
| Ⅱ | 34 | 16 | 18 |  |  |
| Ⅲ | 15 | 8 | 7 |  |  |
| Clincail stage |  |  |  | 1.964 | 0.161 |
| Ⅰ-Ⅱ | 10 | 7 | 3 |  |  |
| Ⅲ-Ⅳ | 44 | 20 | 24 |  |  |
| Tumor size (cm^3^) |  |  |  | 1.405 | 0.495 |
| ≤5 | 7 | 4 | 3 |  |  |
| ＞5 and ≤10 | 11 | 7 | 4 |  |  |
| ＞10 | 36 | 16 | 20 |  |  |
| Invasion into lymp |  |  |  | 4.523 | **0.033*** |
| Yes | 15 | 11 | 4 |  |  |
| No | 39 | 16 | 23 |  |  |
| Distant metastasis |  |  |  | 4.418 | **0.036*** |
| Yes | 10 | 8 | 2 |  |  |
| No | 44 | 19 | 25 |  |  |

*, *P* < 0.05.
